# Pregnancy outcomes in gestational diabetes mellitus: Ethnic‐specific differences among a diverse Asian American population

**DOI:** 10.1002/pmf2.70088

**Published:** 2025-08-17

**Authors:** Candace Levian, Mariam Naqvi, Alexandra Navarrete, Mona Garcia, Tania F. Esakoff

**Affiliations:** ^1^ Department of Obstetrics and Gynecology Cedars‐Sinai Medical Center Los Angeles California USA

**Keywords:** gestational diabetes, glycemic control, insulin, obesity, racial disparities

## Abstract

**Introduction:**

The prevalence of gestational diabetes mellitus (GDM) is higher in Asian populations. Comparative data on pregnancy outcomes among specific ethnic groups within Asian cohorts are lacking. Our objective was to study Asian patients with GDM and describe perinatal outcomes by country of origin.

**Methods:**

Retrospective cohort study of Asian patients with GDM stratified by region of origin (East: China, Korea, Japan, Taiwan; Southeast: Philippines, Thailand, Vietnam; South: India) who delivered at a single institution from 2020 to 2023. Classification of race/ethnicity was self‐reported. Comparisons by geographic subgroup were performed using chi‐square tests. Multivariable logistic regression was then performed to adjust for potential confounders.

**Results:**

We identified 667 patients with GDM during the study period, of whom 274 (41%) identified as Asian (36% East, 32% Southeast, 32% South). Pre‐pregnancy obesity was more likely among Southeast and South Asian patients compared to those from Eastern regions. South Asian patients were more likely to require insulin for blood glucose management and to develop suboptimal glycemic control, defined as failure to meet recommended glycemic targets > 50% of the time despite medical therapy. Southeast Asian patients were more likely to require rapid‐acting insulin. These associations remained significant after adjustment for obesity. Other maternal and neonatal outcomes were similar between groups.

**Conclusion:**

There are differences in glycemic management needs between Asian ethnic groups. Though Asian race is a known risk factor for GDM, patients who identify as Asian represent a heterogeneous group with diverse backgrounds and different clinical outcomes.

## 1 INTRODUCTION

Gestational diabetes mellitus (GDM) affects 8% of pregnancies in the United States with an estimated prevalence of approximately 14% worldwide [1, 2]. GDM is a growing global health concern and while rates are increasing across all racial and ethnic groups, the prevalence of the disease remains disproportionately higher among Asian populations [3]. Recognizing this association, The American College of Obstetricians and Gynecologists identifies individuals of Asian descent as a high‐risk group for the development of GDM [4].

The higher rates of GDM observed among Asian individuals are not fully understood, though factors such as increased insulin resistance, variations in body composition, and dietary patterns have been suggested [5, 6]. General consequences of abnormal glucose tolerance in pregnancy include increased risks of hypertensive disorders, large for gestational age (LGA), macrosomia, and cesarean delivery [4, 7]. Studies suggest Asian patients with GDM may have lower rates of LGA and macrosomia compared to other racial groups, highlighting a difference in clinical outcomes by ancestry [8, 9].

While Asian background is a well‐established risk factor for the development of GDM, this classification encompasses a large, diverse population and comparative data on pregnancy outcomes among specific ethnic groups within Asian cohorts are lacking. The objective of this work was to study Asian patients with GDM and to describe perinatal outcomes by country of origin.

## 2 MATERIALS AND METHODS

This was a retrospective cohort study of Asian American patients with GDM who delivered at a single institution in the United States between January 2020 and December 2023. Patients were stratified by country of origin within Asia: East (China, Japan, Korea, Taiwan), Southeast (Philippines, Thailand, Vietnam), or South (India). Classification of race and ethnicity was self‐reported. Cases of GDM were identified using an obstetric ultrasound database query of the term “gestational diabetes mellitus.” Once identified, data were abstracted through review of electronic health records. This study was approved by the Institutional Review Board.

Patients at this institution were routinely screened for GDM between 24 and 28 weeks gestation via a two‐step approach. A 1‐h, 50‐g glucose challenge test (GCT) was performed and those with a blood glucose level between 140 and 199 mg/dL subsequently took the 3‐h, 100‐g oral glucose tolerance test (OGTT). Patients meeting Carpenter–Coustan diagnostic thresholds were then diagnosed with GDM [10]. A 1‐h GCT result ≥ 200 mg/dL was considered screen‐positive for GDM and these patients did not undergo 3‐h OGTT. Individuals who could not tolerate oral glucose testing (e.g., due to prior bariatric surgery) were instructed to self‐monitor blood glucose levels at home for one week [11]. A diagnosis of GDM was made if more than half of their recorded glucose values were elevated. Overweight and obese patients with additional risk factors underwent early screening for pre‐gestational diabetes followed by repeat screening between 24 and 28 weeks if initial results were normal [4, 12].

All patients with GDM were managed by a multidisciplinary team of obstetricians, maternal‐fetal medicine providers, and certified diabetes care and education specialists. Target blood glucose levels were defined as fasting values < 95 mg/dL and 1‐h postprandial values < 140 mg/dL in over 50% of all recorded measurements. Insulin therapy was prescribed if glycemic goals were unmet through nutrition and exercise modifications alone. The standard approach involved administration of intermediate‐acting insulin (e.g., NPH) to address elevated fasting plasma glucose levels, rapid‐acting insulin (e.g., lispro) for postprandial glycemic control, or a combination of both as clinically indicated.

Both patient demographics and pregnancy outcomes were studied. Blood glucose logs were retrospectively reviewed in the electronic health record to determine level of glycemic control, with suboptimal control defined as failure to meet recommended glycemic targets more than half the time despite medical therapy at any point in the gestation. Other perinatal outcomes, including polyhydramnios (amniotic fluid index ≥ 24 cm or deepest vertical pocket ≥ 8 cm) and LGA (birthweight > 90th percentile for gestational age) were also examined. A composite adverse neonatal outcome was described and included 5‐min Apgar score < 7, hypoglycemia, hypocalcemia, respiratory distress syndrome, hyperbilirubinemia, polycythemia, or death. Neonatal hypoglycemia and hyperbilirubinemia were defined in accordance with guidelines from the American Academy of Pediatrics [13, 14].

Data were summarized by counts and proportions. Comparisons between Asian subgroups were performed using Pearson's chi‐square test with a *p* value of < 0.05 considered statistically significant. To account for the influence of pre‐pregnancy obesity (body mass index ≥ 30 kg/m^2^) as a potential confounder, logistic regression was performed with the East Asian population serving as the reference group. All statistical analyses were performed using StataSE software (version 18).

## 3 RESULTS

**FIGURE 1:**
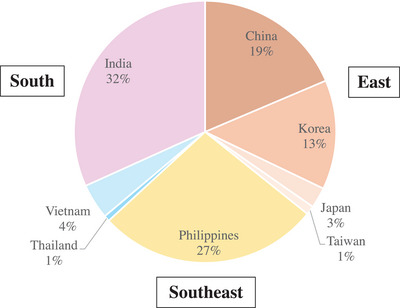
Ethnic groups by Asian region among patients with gestational diabetes mellitus at a single institution. Among patients with GDM who identified as Asian, 36% were from the East [China (51), Korea (37), Japan (7), Taiwan (3)], 32% from the Southeast [Philippines (75), Thailand (2), Vietnam (12)], and 32% from the South [India (87)].

There were 667 patients with GDM during the study period, of whom 274 (41%) identified as Asian (36% East, 32% Southeast, 32% South). Specific countries of origin within each subgroup are shown in the Figure 1.

Baseline patient characteristics are summarized in Table 1. Pre‐pregnancy obesity was more common in Southeast (27%) and South (26%) Asian patients compared to those from Eastern (11%) regions (*p *= 0.01). Rates of advanced maternal age (≥ 35 years), nulliparity, in vitro fertilization, and medical comorbidities including pre‐existing hypertension were similar between the three groups. Personal history of GDM in a prior pregnancy and family history of diabetes mellitus in a first‐ or second‐degree relative were also similar across regions.

**TABLE 1:** Baseline characteristics of Asian patients with GDM stratified by region.

Characteristic	East (*n* = 98)	Southeast (*n* = 89)	South (*n* = 87)	*p*
Maternal age ≥ 35 years	73 (74)	62 (70)	56 (64)	0.33
Pre‐pregnancy BMI ≥ 30 kg/m^2^	11 (11)	24 (27)	23 (26)	0.01
Nulliparous	58 (59)	47 (53)	52 (60)	0.58
In vitro fertilization	20 (20)	12 (13)	10 (11)	0.21
Chronic hypertension	3 (3)	7 (8)	5 (6)	0.35
Thyroid disease	11 (11)	6 (7)	8 (9)	0.57
Prior GDM	17 (17)	13 (15)	15 (17)	0.85
Family history of DM	47 (48)	55 (62)	43 (49)	0.12

*Note*: Data are *n* (%).

Abbreviations: BMI, body mass index; DM, diabetes mellitus; GDM, gestational diabetes mellitus.

Individuals from Southeast (37%) and South (46%) Asia were more likely to require insulin compared to East Asia (20%) (*p *< 0.01), Table 2. Those from South Asia (23%) were also more likely to develop suboptimal glycemic control despite medical therapy when compared to East (9%) and Southeast (16%) Asian patients (*p *= 0.04). Among those receiving insulin for blood glucose management, Southeast Asian patients were most likely to require rapid‐acting formulations with meals and least likely to require intermediate‐acting insulin compared to other Asian regions. Those from Southeast (30%) Asia were also more likely to develop a hypertensive disorder of pregnancy compared to East (19%) and South (15%) Asian countries (*p *= 0.04). There were no statistically significant differences in rates of preterm birth, mode of delivery, shoulder dystocia, or other pregnancy outcomes. The composite adverse neonatal outcome, LGA, and admission to the neonatal intensive care unit (NICU) were similar across Asian regions.

**TABLE 2:** Perinatal outcomes among Asian patients with GDM.

Outcome	East (n = 98)	Southeast (n = 89)	South (n = 87)	*P*
Insulin requirement	20 (20)	33 (37)	40 (46)	< 0.01
Insulin type				
Rapid‐acting	1/20 (5)	6/33 (18)	0/40 (0)	0.01
Intermediate‐acting	19/20 (95)	20/33 (61)	33/40 (82)	< 0.01
Both	0/20 (0)	7/33 (21)	7/40 (18)	0.09
Suboptimal glycemic control^a^	9 (9)	14 (16)	20 (23)	0.04
Delivery < 37 weeks	7 (7)	14 (16)	15 (17)	0.09
Mode of delivery				0.38
Spontaneous vaginal	60 (61)	54 (61)	46 (53)	
Operative vaginal	7 (7)	2 (2)	5 (6)	
Cesarean	31 (32)	33 (37)	36 (41)	
Hypertensive disorder of pregnancy	19 (19)	27 (30)	13 (15)	0.04
Polyhydramnios	1 (1)	2 (2)	5 (6)	0.15
Shoulder dystocia	2 (2)	0 (0)	1 (1)	0.41
Estimated blood loss > 1 L	9 (9)	4 (5)	5 (6)	0.40
Neonatal outcomes				
Composite adverse neonatal outcome^b^	44 (45)	43 (48)	45 (52)	0.65
LGA	3 (3)	4 (5)	6 (7)	0.47
NICU admission	11 (11)	16 (18)	20 (23)	0.10

*Note*: Data are *n* (%).

Abbreviations: GDM, gestational diabetes mellitus; LGA, large for gestational age; NICU, neonatal intensive care unit.

^a^ Suboptimal glycemic control defined as failure to meet recommended glycemic targets > 50% of the time despite medical therapy.

^b^ Composite adverse neonatal outcome consists of 5‐min APGAR < 7, hypoglycemia, hypocalcemia, respiratory distress syndrome, hyperbilirubinemia, polycythemia, or death.

In a logistic regression adjusting for pre‐pregnancy obesity, Southeast (aOR 2.1, 95% CI 1.1–4.1) and South (aOR 3.0, 95% CI 1.6–5.9) Asian patients remained more likely to require insulin for blood glucose management compared to East Asian patients, Table 3. South Asian patients also remained more likely to develop suboptimal glycemic control after adjustment (aOR 2.5, 95% CI 1.1–6.0).

**TABLE 3:** Logistic regression for pregnancy outcomes among Asian patients with GDM.

	Southeast		South	
Outcome	Adjusted^*^ OR (95% CI)	*p*	Adjusted^a^ OR (95% CI)	*p*
Insulin requirement	2.1 (1.1–4.1)	0.03	3.0 (1.6–5.9)	< 0.01
Insulin: rapid	4.2 (0.47–37.9)	0.20	1.0	
Insulin: intermediate	0.07 (0.01–0.62)	0.02	0.22 (0.03–2.0)	0.18
Suboptimal glycemic control	1.7 (0.69–4.3)	0.24	2.5 (1.1–6.0)	0.04
Hypertensive disorder of pregnancy	1.7 (0.85–3.4)	0.13	0.60 (0.27–1.3)	0.21

*Note*: Reference group: East Asian.

Abbreviations: CI, confidence interval; GDM, gestational diabetes mellitus; OR, odds ratio.

^a^ Adjusted for pre‐pregnancy obesity.

## 4 DISCUSSION

This retrospective study contributes data on the varied clinical outcomes observed within subgroups of Asian patients with GDM, a population often treated as a uniformly high‐risk group. Our study found that individuals from Southeast and South Asian regions were more likely to require insulin therapy in the management of GDM and that despite medical therapy, South Asian patients were least likely to achieve optimal glycemic control. This work underscores the need for personalized care in GDM management, demonstrating the limitations of a one‐size‐fits‐all approach.

Our findings are consistent with previously published work examining ethnic‐specific differences in the evaluation and management of GDM. A review of over 3 million births found a higher prevalence of GDM among Asian and Pacific Islander (API) individuals in the United States compared to other racial and ethnic groups. Further analysis revealed significant variations in GDM prevalence within API subgroups, highlighting the need for disaggregated data in health research [15]. A Hawaii‐based study investigating adverse perinatal outcomes in API subgroups with GDM found a higher prevalence of macrosomia in neonates born to Pacific Islander and Filipino individuals compared to neonates born to patients of Japanese, Chinese, and Caucasian descent [16]. Similarly, an Australian study retrospectively reviewed clinical outcomes among five ethnic groups with GDM, including Southeast Asian, South Asian, Middle Eastern, Anglo‐European, and Pacific Islander. Authors found that neonates of South Asian patients with GDM had the lowest birthweights compared to other ethnicities [6]. These clinical differences again demonstrate the limitations of general racial classifications and emphasize the need for ethnic‐specific strategies to address health disparities.

Strengths of our study include the diverse population seen within the Asian cohort of patients with GDM at our institution. Our method of chart review also provided granular data on patient characteristics and outcomes, including in depth review of blood glucose logs. The use of self‐reported race and ethnicity, including countries of origin, facilitated detailed subgroup analysis within the broader Asian population and consequently reduced the risk of misclassification bias.

A limitation of this study is the use of geographically defined subgroups (East, Southeast, and South Asia). While these categories offer greater specificity than grouping all Asian individuals together, they may still obscure important cultural and clinical differences between individual countries within each region. We were thus unable to assess variations in outcomes among individuals from different countries within each subgroup. The single‐center nature of this study may also limit the generalizability of our findings to other populations and healthcare settings. An additional limitation of this study is the relatively small sample size, which, coupled with the infrequent incidence of some of the secondary outcomes such as LGA, may limit the power to detect statistically significant associations for less common events.

Our study uniquely examined GDM outcomes among Asian American patients, disaggregating data by country of origin and enabling a three‐way comparison between individuals from East, Southeast, and South Asian regions. We identified subgroups of patients within the larger Asian classification who ultimately were more likely to require medical therapy for blood glucose management and who were unable to achieve optimal glycemic control despite pharmacotherapy. We also found variations in specific insulin formulations required. These findings are likely explained by global differences in genetic makeup, culture, diet, and lifestyle, further supporting the heterogeneity of this population. For example, dietary patterns high in refined carbohydrates, which are more common in some South Asian cuisines, may contribute to increased insulin resistance [17].

While adverse maternal and neonatal outcomes did not vary significantly by Asian subgroup, our findings suggest that not all patients within a population are alike. Recognizing the complex interplay of factors influencing GDM outcomes, future research should continue to explore these subgroups to develop more effective, ethnic‐specific counseling and management protocols.

## 5 CONCLUSION

There are key differences in glycemic management needs among Asian ethnic groups with GDM. We found variations in the need for pharmacotherapy and glycemic control when comparing patients from East, Southeast, and South Asian regions. While other adverse outcomes were similar between groups, these findings emphasize the heterogeneity within the broader Asian population and highlight the diverse clinical needs of individuals with GDM.

## CONFLICT OF INTEREST STATEMENT

The authors report no conflicts of interest.
